# Optical Silencing of *C. elegans* Cells with Arch Proton Pump

**DOI:** 10.1371/journal.pone.0035370

**Published:** 2012-05-21

**Authors:** Ayako Okazaki, Yuki Sudo, Shin Takagi

**Affiliations:** Division of Biological Science, Graduate School of Science, Nagoya University, Nagoya, Japan; Tokyo Medical and Dental University, Japan

## Abstract

**Background:**

Optogenetic techniques using light-driven ion channels or ion pumps for controlling excitable cells have greatly facilitated the investigation of nervous systems *in vivo*. A model organism, *C. elegans*, with its small transparent body and well-characterized neural circuits, is especially suitable for optogenetic analyses.

**Methodology/Principal Findings:**

We describe the application of archaerhodopsin-3 (Arch), a recently reported optical neuronal silencer, to *C. elegans*. Arch::GFP expressed either in all neurons or body wall muscles of the entire body by means of transgenes were localized, at least partially, to the cell membrane without adverse effects, and caused locomotory paralysis of worms when illuminated by green light (550 nm). Pan-neuronal expression of Arch endowed worms with quick and sustained responsiveness to such light. Worms reliably responded to repeated periods of illumination and non-illumination, and remained paralyzed under continuous illumination for 30 seconds. Worms expressing Arch in different subsets of motor neurons exhibited distinct defects in the locomotory behavior under green light: selective silencing of A-type motor neurons affected backward movement while silencing of B-type motor neurons affected forward movement more severely. Our experiments using a heat-shock-mediated induction system also indicate that Arch becomes fully functional only 12 hours after induction and remains functional for more than 24 hour.

**Conclusions/Sgnificance:**

Arch can be used for silencing neurons and muscles, and may be a useful alternative to currently widely used halorhodopsin (NpHR) in optogenetic studies of *C. elegans*.

## Introduction

Optogenetics is a novel technique for controlling excitable cells such as neurons, muscles and neuroendocrine cells. By exploiting light-driven ion channels or ion pumps derived from microbes and lower eukaryotes, optogenetics enables repression and activation of cells when illuminated with a particular wavelength of light. By enabling temporal manipulation of neuronal activity through an easy-to-induce and non-invasive treatment, this technique has the potential to revolutionize research on the subject of excitable cells *in vivo* [1]–[3].

A nematode, *Caenorhabditis elegans* (*C. elegans*) has a well-characterized neural anatomy: the complete structure as well as the wiring patterns of each neuron of this organism have been determined by reconstruction from serial electron micrograph sections [4]. Together with the availability of various easy-to-handle genetic tools, these characteristics make this model organism an attractive one for studying the functions of single targeted cells in neural networks [5]. On the other hand, the small size of *C. elegans* neurons and their poor accessibility with respect to the application of microelectrodes has hindered conventional electrophysiological approaches. Methods for investigating neural functions in *C. elegans* have thus been almost entirely limited to laser killing of neurons, administration of neuroactive drugs, or use of neural mutants. As neither temporary activation nor repression of neuronal activity can be achieved with traditional techniques such as laser killing or drug treatment, optogenetic methods will be of great benefit to the study of *C. elegans* neurons. Optogenetics allows the investigation of neuronal functions in freely moving animals, increasing the prospects for behavioral studies using *C. elegans*. Optogenetic approaches applied to *C. elegans*, using channelrhodopsin-2 for activation of cells, and halorhodopsin (NpHR) for silencing of cells, have been reported [6]–[8].

NpHR is a light-driven inward chloride pump present in *Natronomonas pharaonis* [9], which inhibits electrical excitation of genetically targeted cells under illumination by yellow light (580 nm) with millisecond temporal resolution. NpHR, however, is capable of only modest hyperpolarizing currents in response to light, and once activated, NpHR enters long-lasting inactive states [10]. Recently, an optogenetical application of another light-driven pump, Archaerhodopsin-3 (Arch), derived from halobacteria *halorubrum sodomense*, was reported [11]. Arch is an outward proton pump driven by green light (550 nm), and generates a neural silencing current at a lower level of illumination *in vitro* compared with NpHR. Additionally, Arch recovers spontaneously from light-dependent inactivation. It has been reported that Arch enables the silencing of neurons in the awake brain of a mouse [11]. Therefore, Arch may serve as another useful optogenetic tool for neural silencing experiments *in vivo.*

In this study, we examined the applicability of Arch for optogenetic manipulation of *C. elegans* cells *in vivo*. We found that worms forced to express Arch::GFP in neurons or muscle cells of the entire body stopped their locomotion and became paralyzed when illuminated with green light. Worms expressing Arch::GFP in different subsets of motor neurons exhibited distinct behavioral defects under green light illumination. We also examined the acquisition of Arch function and its stability in *C. elegans* cells by using a heat-shock gene induction system. We found that Arch becomes fully functional 12 hours after induction of expression, and remains functional for at least 24 hours.

## Results

### Arch::GFP Expression in *C. elegans* Cells

To examine whether Arch, a light-driven proton pump, can be used to repress the electrical activity of neurons and muscle cells in *Caenorhabditis elegans* (*C. elegans*), we first tried to express C-terminally GFP-tagged Arch (Arch::GFP) in worms by using the *myo-3* gene promoter, which encodes a myosin heavy chain expressed specifically in the body wall and vulva muscles of the organism, and the promoters of the *F25B3.3* and *aex-3* genes, both of which drive pan-neuronal expression. Then, we analyzed the expression pattern of GFP and the locomotion behavior of transgenic worms under green light illumination.

**Figure 1:**
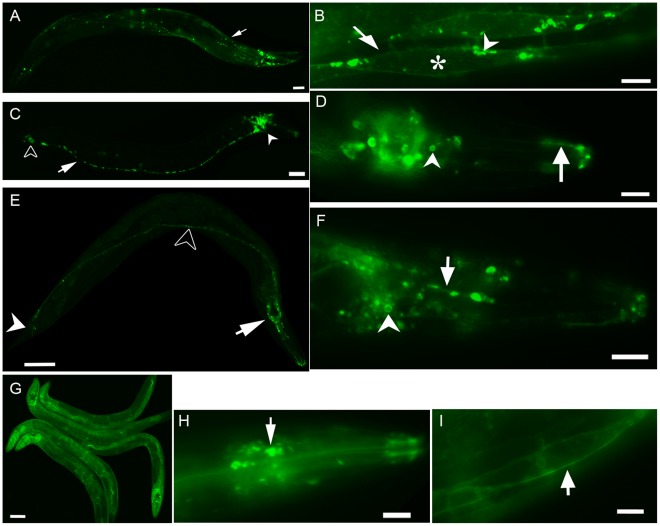
Expression of Arch::GFP in *C. elegans* driven by various promoters. (A) A fluorescent micrograph of an *nc3031Ex[myo-3p::Arch::gfp]* animal. Arch::GFP is expressed in longitudinal bands composed of body wall muscles (arrow). (B) An enlarged view of body wall muscle cells. GFP signal is observed along the outline of muscle cells (arrow). Vesicular structures visualized with GFP are localized close to the cell membrane (arrow head). Weak GFP signal is detected in the cytoplasm (asterisk). (C) Expression of Arch::GFP in an *nc3034Ex[F25B3.3p::Arch::gfp]* animal. Arch::GFP is expressed in head neurons (arrow head), tail neurons (open arrow head) and the ventral nerve cord (arrow). (D) The head of an animal carrying *F25B3.3p::Arch::gfp*. Arch::GFP is expressed in the axon (arrow) and the cell body (arrow head) of head neurons. (E) Expression of Arch::GFP in an *nc3026Ex[aex-3p::Arch::gfp]* animal. Arch::GFP is expressed in head neurons (arrow), tail neurons (arrow head), and the ventral nerve cord (open arrow head). (F) The head of an animal carrying *aex-3p::Arch::gfp*. Arch::GFP is expressed in the axon of head neurons in a punctured pattern (arrow). GFP is seen on the cell membrane of a cell body (arrow head). (G) Expression of Arch::GFP in an *nc3003Ex[hsp-16.2p::Arch::gfp]* animal. Arch::GFP is expressed everywhere in the body. (H) The head of an animal carrying *hsp-16.2p:: Arch::gfp*. Arch::GFP is expressed in neurons (arrow). (I) Body wall muscle of an *hsp-16.2p::Arch::gfp* animal. GFP is clearly localized at the cell membrane (arrow). Scale bar: A, C, E, G = 100 µm; B, D, F, H I = 10 µm. Anterior is toward the right except for (G).

In worms carrying the transgene *myo-3p*::*Arch*::*gfp*, *nc3031Ex[myo-3p::Arch::gfp]*, an intense GFP signal was observed in granular or vesicular structures close to the cell membrane and in myofibril-like structures of the body wall muscles. The signal was also detected at muscle cell boundaries, suggesting localization of Arch to the cell membrane. A weak GFP signal was seen in the cytoplasm, but not in the nucleus (Fig. 1A, B).

In worms carrying the transgene *F25B3.3p::Arch::gfp*, *ncEx3034[F25B3.3p::Arch::gfp]*, and in worms carrying the transgene *aex-3p::Arch::gfp, ncEx3026[aex-3p::Arch::gfp]*, GFP signals were observed in the soma and the axon of many neurons in the head, mid body and tail (Fig. 1C, E). The signal often demarcated the cell soma. An intense granular or vesicular GFP signal was sometimes detected in the soma and in axons (Fig. 1 D, F).

All transgenic strains carrying *Arch::gfp* stopped locomotion and became paralyzed under green light illumination to the whole body. *C. elegans* worms do not have inherent rhodopsin genes [12], and it is presumed that the level of endogenous retinoids, if these even exist, is insufficient for the proper functioning of rhodopsins [6]. Accordingly, when the transgenic animals were raised in the absence of all-trans-retinal (ATR), an essential cofactor for rhodopsin, illumination with green light failed to stop their locomotion, proving that the paralysis elicited in the presence of ATR is indeed driven by Arch.

### Light Intensity Dependence of Arch Neuronal Silencing Activity

**Figure 2:**
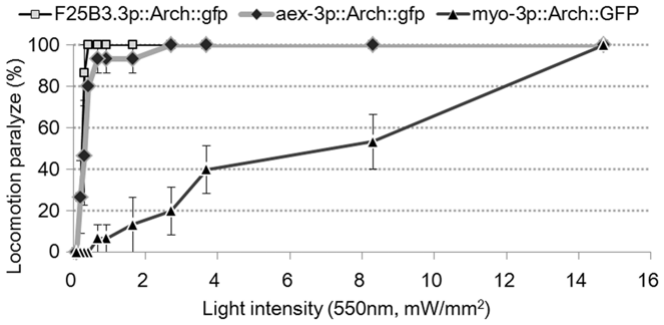
Dependence of locomotion paralysis on light intensity. Animals expressing Arch::GFP under muscle-specific (*myo-3p*) and pan-neuronal (*aex-3p* and *F25B3.3p*) promoters were illuminated with green light at varying intensities. Animals with *nc3034Ex[F25B3.3p::Arch::gfp]* (squares) and *nc3026Ex[aex-3p::Arch::gfp]* (diamonds) exhibited higher responsiveness at lower light intensities than *nc3031Ex[myo-3p::Arch::gfp]* (triangles) animals did. (mean±SEM; n = 3. Five animals were examined for each trial.).

We then examined the response of each transgenic line expressing Arch::GFP in neurons to green light of different intensities. With *ncEx3034[F25B3.3p::Arch::gfp]* animals expressing Arch::GFP pan-neuronally, 25% of the worms were paralyzed when illuminated with green light (542.5±67.5 nm) with an intensity of 0.2 mW/mm^2^ at 550 nm, the Arch absorption maximum. Eighty-five percent of the animals stopped locomotion at 0.3 mW/mm^2^, and all animals stopped locomotion at 0.4 mW/mm^2^ (Fig. 2). Twenty-five percent of the *ncEx3026[aex-3p::Arch::gfp]* animals, another pan-neuronally Arch::GFP-expressing line, stopped locomotion at 0.2 mW/mm^2^, and 50% of the animals stopped locomotion at 0.3 mW/mm^2^. At 2.7 mW/mm^2^, all animals stopped locomotion (Fig. 2).

*ncEx3034[F25B3.3p::Arch::gfp]* worms stopped locomotion immediately upon exposure to green light at 14.7 mW/mm^2^, remained paralyzed during the illumination for 5 seconds, and resumed normal locomotion immediately after the end of such illumination, suggesting that Arch activation and inactivation both occur very rapidly in response to illumination conditions in this case. The observed quick response to conditions of illumination and non-illumination was almost fully retained even when worms were subject to 10 cycles consisting of a 1 second period of illumination followed by 1 second without illumination. When subjected to 30 cycles in this manner, however, the recovery of locomotion after illumination was turned off gradually required more time for most worms. When subjected to continuous illumination for 30 seconds, most *ncEx3034[F25B3.3p::Arch::gfp]* worms (80%, n = 60) remained paralyzed throughout the illumination, but the others started movement, though without forward or backward locomotion, while under illumination. Thus, continuous illumination appears to have a relatively minor inactivating effect on Arch. Post-illumination recovery was delayed in the majority of worms (83%, n = 60), but they eventually resumed locomotion within 15 seconds after illumination was stopped.

### Light Intensity Dependence of Arch Muscle Silencing Activity

In a manner similar to that for the observed Arch neuronal silencing activity, the locomotion of *ncEx3031[myo-3p::Arch::gfp]* animals expressing Arch in body wall muscle cells was profoundly affected by green light illumination. With *ncEx3031[myo-3p::Arch::gfp]* animals, however, illumination did not lead to immediate arrest of locomotion; worm locomotion first slowed down, and paralysis gradually occurred. At an illumination intensity of 14.7 mW/mm^2^, complete arrest of locomotion required illumination for 8 seconds (n = 9). This is in contrast to worms expressing Arch in neurons, which stopped locomotion immediately after the onset of illumination. Therefore, we evaluated the effect of illumination on the locomotion of *ncEx3031[myo-3p::Arch::gfp]* animals over 30 seconds of illumination. At 0.7 mW/mm^2^, only a fraction of the animals had stopped locomotion. At 8.3 mW/mm^2^, approximately half of the animals stopped locomotion, whereas all animals appeared to be paralyzed when subjected to 14.7 mW/mm^2^ illumination (Fig. 2).

When continuously exposed to green light at an intensity of 14.7 mW/mm^2^ for a period of 30 seconds, some *ncEx3031[myo-3p::Arch::gfp]* animals remained paralyzed during illumination (48% n = 55), but others recovered partially and started to move while still under illumination, albeit at a much slower rate than usual. With an illumination period longer than 30 seconds, or when subjected to intermittent illumination cycles consisting of 1 second of illumination followed by 1 second of non-illumination, repeated 30 times, locomotion recovery required more time. Some worms failed to resume normal fast locomotion even after a 10-second period of non-illumination. Given a sufficient time following light cessation, however, all worms eventually resumed normal locomotion.

### Light-mediated Silencing of Subsets of Ventral Cord Motor Neurons

In order to utilize Arch for analyzing the function of neuron-circuits of worms, we then tried to express Arch in subsets of neurons. Freely moving *C. elegans* worms crawl forward on agar in a sinusoidal motion, which was occasionally interrupted by brief backward movements. Worms can also be directed to move backward or forward by tactile stimulation. We have examined how locomotory behaviors are affected by silencing different subsets of motor neurons in the ventral nerve cord. The locomotion is executed by body wall muscles whose contraction and relaxation is regulated by the cholinergic A- and B-type motor neurons, and the GABAergic D-type motor neurons, respectively [13], [14], [15]. Previous studies also showed that A-type motor neurons (VA, DA) direct backward locomotion while B-type motor neurons (VB, DB) direct forward locomotion [13], [16].

**Figure 3:**
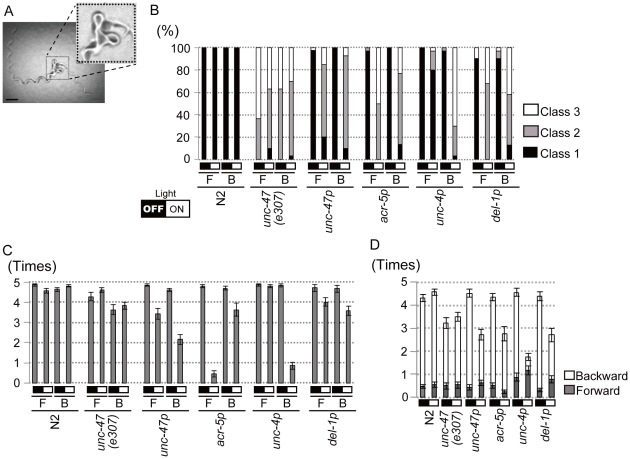
Defects in the locomotory behavior caused by silencing subsets of motor neurons. (A) A crawling track of an *ncEx2351[unc-47p::Arch::gfp]* animal. When the freely moving animal was illuminated with green light for 1 minute, it performed loopy movement (dotted square). After turning off the green light, it resumed normal sinusoidal movement. Scale bar = 500 µm (B-D) Locomotory behavior of worms expressing Arch in motor neurons subsets. Arch::GFP was expressed in D-type (VD, DD), A-type (VA, DA), B-type (VB, DB) and VA +VB motor neurons in *ncEx2351[unc-47p::Arch::gfp], ncEx2371[acr-5p::Arch::gfp], ncEx3068[unc-4p::Arch::gfp]* and *ncEx2365[del-1p::Arch::gfp]* animals, respectively. Locomotion of *unc-47(e307)* animals was also scored. For all transgenic strains, animals behaviors under green light illumination (ON = open box) and those without illumination (OFF = filled box) differed significantly (p<0.001, Fisher's exact test for the locomotion assay under the freely moving condition and Student's *t* test for the touch response assay). Exceptions are forward movement of *ncEx3068[unc-4p::Arch::gfp]* animals, which did not change significantly under illumination, and forward movement of *ncEx2365[del-1p::Arch::gfp]* animals elicited by gentle posterior touch (p = 0.012). Error bars indicate ±SEM. (B) Forward (F) and backward movement (B) was scored in worms moving freely. Percentage of animals exhibiting the “Class 3 (severe)”, “Class2 (mild)” and “Class1 (no)” phenotype in locomotory behaviors when they were illuminated with green light is shown. Defects were classified as “Class 3″ when no corresponding movement or response was observed. Other abnormalities, such as retardation or decrease in the extent of movement, are classified as “Class2”. (C) Forward movement (F) to gentle posterior touch and backward (B) movement to gentle anterior touch were scored. Responses out of five touches are shown. (D) Forward (F) and backward movement (B) to harsh touch was scored, and was shown additively in each bar. Responses out of five touches are shown.

First, to achieve silencing of D-type motor neurons (VD, DD) selectively, Arch::GFP was expressed under the control of the promoter of the *unc-47* gene encoding a vesicular GABA transporter [17] (Fig. S1A). Without illumination, *ncEx2351[unc-47p::Arch::gfp]* animals moved in a normal sinusoidal motion. When *ncEx2351* animals moving freely were illuminated with green light, they exhibited loopy sinusoidal forward movement (Fig. 3A), and movement of some animals appeared to slow down in either direction. In wild type animals, gentle touch to the anterior part of the body elicits backward movement, whereas posterior touch elicits forward movement. Under green light illumination, the extent of backward movement elicited by gentle anterior touch was smaller in *ncEx2351* worms (Fig. 3C). Likewise, forward movement elicited by gentle posterior touch was affected to various extents. We observed that the onset of touch elicited movement was delayed about 1 second in 2 out of 30 animals, suggesting that the GABAergic system might play a role in switching promptly the direction of movement. We also examined the effect of silencing of GABAergic neurons in the background of Roller phenotype in *ncEx2311[unc-47p::Arch::gfp; rol-6d]* animals. Green light illumination retarded or arrested the locomotion, which lasted over 60 seconds of illumination (Fig. S2).

Slow locomotion and defective responses to gentle touch are the phenotypes observed in mutants with defects in GABA neurons (Fig. 3B, C). A previous study also showed that killing the four GABAergic RME neurons in the head of wild type worms resulted in an abnormal loopy foraging behavior [14], . Thus, green light illumination to *ncEx2351* animals appears to lead to phenotypes expected from silencing GABAergic neurons.

Next, to achieve silencing of A-type motor neurons specifically, Arch::GFP was expressed under the control of the promoter of the *unc-4* gene [19], [20] (Fig. S1B). When illuminated with green light, forward movement of freely moving *ncEx3068[unc-4p::Arch::gfp]* animals was normal, but backward movement was abnormal: Whereas normal worms move backward a distance of about one body length, *ncEx3068[unc-4p::Arch::gfp]* animals often moved backward only as far as a half the body length, and sometimes did not move at all (Fig. 3B). Under green light illumination, anterior gentle touch to *ncEx3068[unc-4p::Arch::gfp]* worms did not elicit backward movement, or did it only incompletely (Fig. 3C). In contrast, the posterior touch elicited normal forward movement. Sometimes worms stopped moving for a while after a harsh touch.

To achieve silencing of B-type motor neurons specifically, the promoter of the *acr-5* gene was used [20] (Fig. S1C). When illuminated with green light, forward movement of *ncEx2371[acr-5p::Arch::gfp]* animals slowed down, and about half of the animals stopped locomotion completely within 10 seconds (Fig. 3B). The locomotion was arrested under continuous illumination over 20 minutes. The posterior half of the body was often motionless, while the anterior part of the body connected to the head moved. Backward movement was also affected, though to a lesser extent, and it often stopped halfway. Posterior gentle touch to *ncEx2371[acr-5p::Arch::gfp]* animals under green light illumination failed to elicit forward movement almost completely (Fig. 3C). Although in most cases animals responded to anterior gentle touch, they retreated a shorter distance and their movement was loopy.

When *ncEx2365[del-1p::Arch::gfp]* animals, which expressed Arch::GFP in VA and VB motor neurons under the control of the *del-1* gene promoter [20], [21] (Fig. S1D), were illuminated with green light, locomotion appeared to slow down in either direction (Fig. 3B). Response to gentle touch was sometimes affected in either direction (Fig. 3C). Backward movement was often incomplete.

### Arch::GFP Expressed by Heat-shock Promoter

Next, to investigate the timing of the acquisition of the silencing function and the stability of Arch following its expression in *C. elegans* cells, we exploited a gene induction system using the promoter of the heat-shock protein gene *hsp-16.2* [22].

In *ncEx3003[hsp-16.2*p*::Arch::gfp]* animals, GFP expression was detected 3 hours after heat-shock. The level of GFP expression then increased gradually, reached a stable plateau 9–12 hours after heat shock, remained constant for as long as 24 hours and started to decay slowly thereafter. The level of GFP expression in most animals 48 hours after heat shock was lower compared with the level 24 hours after heat shock, but higher compared with the level 3 hours after heat shock. In some animals, GFP expression was detected even 72 hours after heat shock.

The GFP signal was observed in the entire region of the body (Fig. 1G). In neurons, the GFP signal was localized to the cell membrane and in the cytoplasm, but not in the nucleus (Fig. 1H). In muscle cells, the GFP signal was high at the cell surface (Fig. 1I). An intense GFP signal was sometimes detected in granular or vesicular structures in the cytoplasm. To help clarify the localization of Arch in cells, we compared Arch expression with the GFP signals in *ncIs17[hsp-16.2*p*::gfp]* transgenic worms, which only express GFP under the control of the same heat-shock promoter [23]. The GFP signal in *ncIs17[hsp-16.2*p*::gfp]* worms was distributed diffusely in the cytoplasm, without visualizing granular or vesicular structures or demarcating cell boundaries. These differences further support the conclusion that Arch::GFP is localized to intracellular vesicles and the cell membrane.

**Figure 4:**
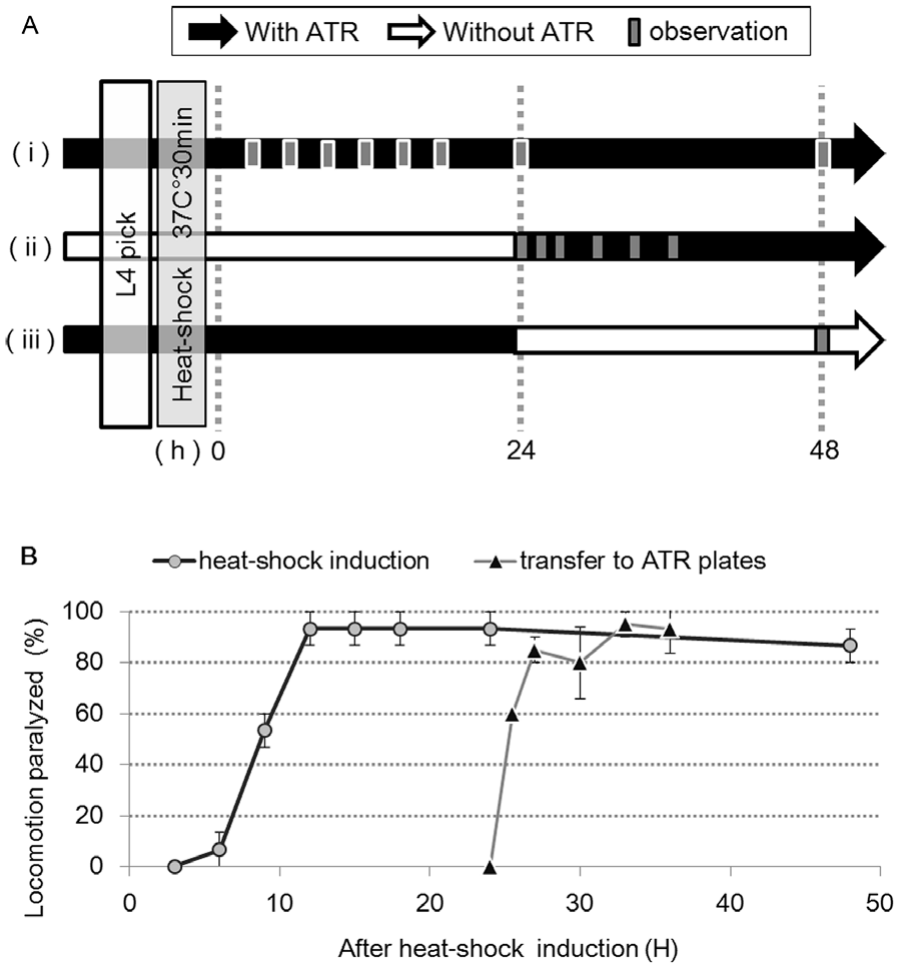
Locomotion assay using heat shock-mediated induction of Arch. (A) Scheme showing the schedule of transferring worms to plates with or without all-trans-retinal (ATR) in heat shock induction experiments. i: Animals were cultivated in the presence of ATR throughout the experiments. (Figs. 3B, 4B) ii: Animals were transferred to ATR-supplemented plates 24 h after heat shock (Fig. 4B). iii: Animals were cultivated in the presence of ATR and transferred to ATR-free plates 24 h after heat shock. (B) Time course of light-elicited locomotory paralysis after heat-shock induction of Arch::GFP. Heat-shocked *nc3003Ex[hsp-16.2p::Arch::gfp]* animals cultivated in the presence of ATR throughout the experiment (circles) (Fig. 4. A(i)) or transferred from ATR-free to ATR-supplemented plates 24 h after heat shock (triangles) (Fig. 4. A(ii)) were examined at each time point. When ATR was present throughout the experiment (circles), paralysis of worms was first noticed 6 h after heat shock. The paralysis rate reached a plateau 12 h after heat shock, and remained constant for 48 h. (mean±SEM; n = 3, Five animals were examined for each trial.) Worms cultivated in the absence of ATR throughout the experiment did not respond to illumination at any time point. When animals were grown and heat shocked in the absence of ATR and then transferred to ATR-supplemented plates 24 h later (triangles), half of them were paralyzed by illumination 1.5 h after transfer, and the paralysis rate reached a plateau within 3 h. (mean±SEM; n = 4, Five animals were examined for each trial.).

Next, we investigated the function of Arch expressed by heat shock (Fig. 4A(i), B). Three hours after heat shock, green light illumination at 19.6 mW/mm^2^ had no effect on the locomotion of heat-shock-induced *ncEx3003[hsp-16.2*p*::Arch::GFP]* worms, although a GFP signal was clearly observed. Six hours after heat shock, 5% of the animals stopped locomotion in response to green light illumination. In response to green light illumination, half of the animals stopped locomotion 9 hours after heat shock, and 90% of the animals stopped 12 hours after heat-shock. Up to 48 hours after heat shock, green light illumination still caused locomotion arrest in most animals. Some animals stopped locomotion in response to green light illumination even 72 hours after heat shock, although the fraction of paralyzed worms (20%, n = 10) was much smaller than the fraction 48 hours after heat shock.

To summarize the temporal changes of the Arch silencing function and expression, the worms became fully responsive to green light illumination 12 hours after heat shock, while Arch expression monitored with GFP reached the maximal level 9–12 hours after heat shock. Thereafter, the responsiveness of worms to green light illumination appeared to correlate with the expression level of Arch::GFP, which sometimes persisted for longer than 48 hours. Since heat-shock-mediated expression of GFP is induced only temporarily, the long-lasting perdurance of GFP expression, as well as the responsiveness to light observed in some worms, indicate that Arch can be stably retained in a functional form for a long period.

We also investigated the light sensitivity of *Ex3003[hsp-16.2*p*::Arch::GFP]* worms 24 hours after heat shock. A small fraction of animals stopped locomotion when illuminated at 2.2 mW/mm^2^, half of the animals stopped at 4.9 mW/mm^2^, and all the animals stopped when illuminated at 11.1 mW/mm^2^.

### Uptake and Stability of ATR in Arch

The heat-shock induction experiments described above suggested that Arch acquire silencing function fully only 12 hours after heat shock induction. The unexpectedly long period that is needed before Arch acquires silencing function prompted us to examine the possibility that Arch may need to undergo maturation processes that might require several hours to complete. To investigate whether ATR, an essential cofactor of Arch, might be involved in the posited maturation processes, we examined the incorporation and stability of ATR in Arch.

We examined whether the time required for incorporation of ATR into Arch caused the observed delay of several hours before Arch becomes functional following its expression (Fig. 4). *ncEx3003[hsp-16.2*p*::Arch::gfp]* worms raised in the absence of ATR were heat shocked and were transferred to a plate containing ATR 24 hours later (Fig. 4A(ii)). The worms raised without ATR expressed GFP similarly to those raised with ATR. About half of the animals stopped locomotion in response to green light illumination 1.5 hours after transfer, and more than 80% of the animals stopped locomotion under green light 3 hours after transfer (Fig. 4B). Therefore, ATR seems to be incorporated into the preexisting Arch that was synthesized in the absence of ATR, and function there. The results also indicate that Arch is stable in the absence of ATR. Importantly, the relatively short 1.5-hour period that worms required before becoming responsive to green light illumination following transfer to ATR-containing plates indicates that incorporation of ATR into Arch occurs rather rapidly, and this alone probably cannot explain the observed delay of several hours.

We also examined how long ATR keeps functioning after being incorporated into Arch. First, we estimated how long the ATR that worms have ingested with food remains available for Arch when worms are transferred to plates without ATR. *ncEx3003[hsp-16.2*p*::Arch::gfp]* worms raised in the presence of ATR were heat shocked and transferred to a plate without ATR at various time points. When transferred immediately or 3 hours after heat shock, worm locomotion was not interrupted in response to green light for at least 48 hours after transfer. Given that the expression level of Arch 9 hours after heat shock is sufficiently high for half the worms to show a response to illumination when ATR is present, this result indicates that the level of ATR in the body of worms after transfer to an ATR-free plate seems to be reduced to a level insufficient to be incorporated into Arch within 6 hours. We then examined the stability of incorporated ATR. *ncEx3003[hsp-16.2*p*::Arch::gfp]* worms raised in the presence of ATR were heat shocked and transferred to a plate without ATR 24 hours later (Fig. 4A(iii)). When exposed to green light illumination, the worms stopped locomotion even 24 hours after transfer (93%, n = 15), when free ATR should be no longer available for Arch in the body of the worms. Therefore, ATR incorporated into Arch appears to be stable and keep functioning for at least 24 hours.

### Effects of GFP Segment in Arch::GFP

Another possible rate-limiting step in the process whereby Arch acquires a silencing function is translocation to the plasma membrane. Although we did not notice any obvious change in the localization pattern of Arch::GFP around 9 hours after heat shock, a slight change in localization, such as a shift to the plasma membrane from nearby cytoplasm, might nevertheless lead to significant functional differences. To investigate whether removing the GFP segment, whose hydrophilic nature can potentially affect incorporation into membranes, from Arch::GFP might facilitate translocation of Arch to the plasma membrane, we generated transgenes expressing Arch alone via a heat-shock promoter, *Ex[hsp-16.2*p*::Arch]*.

**Figure 5:**
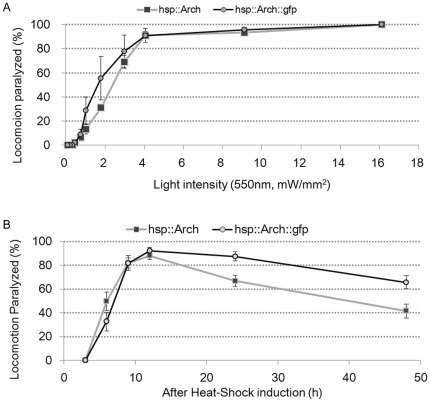
Comparison of light-elicited locomotory paralysis mediated by Arch and Arch::GFP. (A) Dependence of locomotion paralysis of *Ex[hsp-16.2p::Arch]*animals (black squares) and *Ex[hsp-16.2p::Arch::gfp]* animals (open circles) to light intensity was measured 12h after heat shock. (B) Time course of light-elicited locomotory paralysis after heat-shock induction of Arch and Arch::GFP. Heat-shocked *Ex[hsp-16.2p::Arch]* (black squares) and *Ex hsp-16.2p::Arch::gfp]* (open circles) animals were examined at each time point as shown in Fig. 4. A(i). Three independently isolated lines were used for each transgene: *ncEx3002, ncEx3003* and *ncEx3004* for *Ex[hsp-16.2p::Arch::gfp]*, and *ncEx3039, ncEx3040* and *ncEx3041* for *Ex[hsp16-2p::Arch]*. Shown are the mean±SEM of 9 trials consisting of 3 trials for each line. Five animals were examined for each trial.

The dependence of responsiveness to light intensity was almost identical for *Ex[hsp16-2*p*::Arch]* and *Ex[hsp-16.2*p*::Arch::gfp]* animals, with slightly higher responsiveness for the former at lower light intensities (Fig. 5A) (*p*<0.05 Student’s *t*-test). With *Ex[hsp16-2p::Arch]*, 2% of the animals became paralyzed at an illumination intensity of 0.5 mW/mm^2^, half of the animals at 3.0 mW/mm^2^ and all the animals at 16.1 mW/mm^2^. With *Ex[hsp-16.2p::Arch::gfp]*, 2% of the animals became paralyzed at 0.5 mW/mm^2^, half of the animals at 1.8 mW/mm^2^, and all the animals at 16.1 mW/mm^2^.

The responsiveness of *Ex[hsp-16.2*p*::Arch]* animals to light also changed over time in a manner similar to that of *Ex[hsp16-2*p*::Arch::gfp]* animals (Fig. 5B). With *Ex[hsp16-2*p*::Arch]* animals, paralysis by green light illumination was first noticed 6 hours after heat shock and the percentage of paralyzed animals increased thereafter. The worms exhibited maximum responsiveness around 12 hours after heat shock, with 95% of the animals stopping locomotion. Thus, deletion of GFP from Arch::GFP does not appear to accelerate the acquisition of the silencing function. The responsiveness to light after 12 hours decreased slightly faster in *Ex[hsp-16.22*p*::Arch]* animals (*p*<0.01 Student’s *t*-test): 40% of *Ex[hsp-16.2p::Arch]* animals were paralyzed by green light illumination 48 hours after heat shock, while more than 60% of *Ex[hsp-16.2p::Arch::gfp]* animals were paralyzed. It appears that the GFP segment might inhibit the degradation of Arch.

## Discussion

In this paper we report for the first time that Arch derived from archaebacteria can be expressed in *C. elegans* cells without apparent adverse effects, and can be used to silence activity of neurons and muscles, thereby modifying worm’s behaviors.

Arch expressed in neurons and muscles affects worm behavior differently.

We found that illumination at a similar level of light intensity affected worms differently depending on whether Arch was expressed in neurons or muscles. An apparently stronger GFP signal in neurons compared with muscle cells in our transgenic lines suggests that the concentration of Arch::GFP is higher in neurons. Since the amplitude of change in membrane potential driven by Arch-mediated proton current is probably proportional to the density of Arch on the cell membrane, illumination with the identical light intensity would promote larger hyperpolarizing effects in neurons than in muscle.

Neighboring muscle cells are known to be coupled electrically through gap junctions [24]. With their larger cell size, mosaic expression of Arch and electric coupling of cells in muscle tissues would weaken the polarizing effect of the Arch-mediated proton current. *Ex[myo-3p::Arch::GFP]* transgenic lines so far established express Arch::GFP in highly mosaic patterns, and we have noticed the presence of dead embryos expressing GFP very intensely in line cultures. Although we have not noticed any morphological damage in Arch-expressing muscle cells, highly abundant expression of Arch or the presence of excessive amounts of the promoter sequence of the *myo-3* gene in muscle cells might have a harmful effect on the development or survival of worms.

The heat-shock-mediated Arch expression in *Ex[hsp-16.2p::Arch::GFP]* worms occurred ubiquitously. While Arch expressed both in neurons and in muscle cells may affect locomotion, the finding that *Ex[hsp-16.2p::Arch::GFP]* worms exhibited higher sensitivity to green light illumination than *Ex[myo-3::Arch::GFP]* worms suggest that locomotory paralysis in *Ex[hsp-16.2p::Arch::GFP]* worms is mainly attributed to the silencing of neurons rather than muscle cells.

### Arch-expressing *C. elegans* Responds to Light Precisely

By employing various illumination patterns, we showed that worms expressing Arch respond to light quickly and precisely. This finding is consistent with a previous report showing that Arch-mediated neuronal silencing occurs within a millisecond time range [11]. We also showed that continuous illumination for as long as 30 seconds leads to sustained paralysis in most worms expressing Arch in neurons, which is also consistent with a previous finding that prolonged activation does not significantly affect Arch activity.

Since Arch is an outward proton pump [11], prolonged activation of Arch may cause crucial changes in intra- and extracellular pH levels. The failure of Arch-expressing worms to immediately recovery locomotion after the cessation of illumination following prolonged illumination might be due to disturbances of cellular pH conditions. Nevertheless, the absence of long-lasting effects on neurons or muscle cells is notable, since worms eventually resumed locomotion at normal rates in the absence of illumination.

Silencing of different subsets of neurons elicited distinct behavioral defects.

To investigate the neuron-circuits governing the locomotory behavior of worms, we have used several promoters that drive expression of Arch in selected subsets of motor neurons of the ventral nerve cord. We found that worms expressing Arch by different promoters exhibited distinct behavioral phenotypes specific for each promoter when illuminated with green light.

Responses of worms expressing Arch in GABAergic neurons including D-type motor neurons generally mimic the phenotype of genetic mutants defective in functioning of GABAergic neurons, supporting the notion that specific silencing of GABAergic neurons is achieved successfully. We have, however, observed several differences in the effects between acute silencing and chronic inactivation in mutants. Under a freely moving condition, animals expressing Arch in GABAergic neurons moved more actively than *unc-47* mutants. In *unc-47* mutants, movement of the head and tail are usually repressed, while green light-mediated silencing of GABAergic neurons elicited enhanced head movement, which resulted in loopy forward movement. We also noticed that light-mediated silencing of GABAergic neurons affected touch responses more strongly than a mutation in the *unc-47* gene. The differences may reflect in part incomplete silencing of the GABAergic system due to the mosaic expression of Arch, which is characteristic for transgenes carried by an extrachromosomal array. It may be also due to the properties of the particular mutant allele used in this study. Alternatively, although the finding that acute silencing and chronic inactivation generally has similar effects suggests the absence of developmental mechanisms acting to compensate for locomotory defects of the mutants, neuron-circuits relevant to certain behaviors might be modified through chronic inactivation of GABAergic neurons.

We found that silencing subsets of cholinergic motor neurons had neuron subset-specific effects. At hatching, only three types of motor neurons are present, DA, DB and DD. A classic study using the laser ablation method showed that killing most of DA in newly hatched larvae resulted in animals that could not move backward but could move forward normally, whereas killing most of DB neurons resulted in defective forward movement without affecting backward movement [16]. Light-mediated activation of Arch expressed in DA and VA motor neurons by using the *unc-4* promoter caused the direction-specific defect in locomotion, confirming the essential role of A-type neurons in backward movement. The results of the Arch expression experiment using the *arc-5* promoter, which drives gene expression in DB and VB motor neurons, but not in A-type motor neurons, also confirmed the essential role of B-type motor neurons in forward movement. Interestingly, we have found that backward movement was often performed incompletely by silencing B-type neurons. While B-type neurons may not play a significant role in triggering backward movement, they might be critically required during the later phase of backward movement. By silencing both VA and VB neurons in *ncEx2365[del-1p::Arch::gfp]* animals, we found that movement in either direction was affected. Accordingly, a previous study showed that *mec-4(gf)-*mediated genetic cell ablation under the control of the *del-1* promoter resulted in uncoordinated movement, in which either the forward or backward was irregular and appeared to be executed with difficulty [21].

To summarize, behavioral phenotypes elicited by silencing each subset of motor neurons are generally consistent with known functions of the corresponding neuron subset. It should be noted that we have used the transgenes carried by extrachromosomal arrays, which lead to mosaic expression of Arch among target neurons, indicating that complete silencing of a targeted neuron subset would result in a stronger and clearer phenotype. It is also noted that the behavioral changes were observed throughout the period of continuous illumination, which was sometimes longer than 20 minutes, further confirming the utility of Arch as an optogenetic tool in *C. elegans*.

### Time Lag Between Induction and Functioning of Arch

The induction of Arch by heat shock seems to occur only transiently. It was reported that the level of *hsp16-2* mRNA reached a maximum 1 hour after onset of heat shock, and decreased to the normal level within 2 hours after heat shock was terminated [22]. We found that the expression level of Arch::GPF did not increase markedly later than 9 hours after heat shock, indicating that synthesis of Arch persisted only for a limited period. This transient induction system enabled us to reveal hitherto unnoticed properties of Arch *in vivo*.

We found that Arch remains functional for a relatively long time in *C. elegans* cells, sometimes for several days, which is one advantage that Arch has as an experimental tool. An unexpected finding is that Arch becomes fully functional only 12 hours after its induction. We also found that the expression of Arch::GPF first detected 3 hours after heat shock and the expression level increased up to 9 hours after heat shock. This relatively slow onset and gradual increase in Arch::GPF expression is likely to account for a major part of the observed delay in acquiring a silencing function after Arch induction. It is, however, possible that other processes, such as translocation to the cell membrane and molecular modification, may also partly contribute to the delay.

To affect membrane potential effectively, Arch must be recruited to the cell membrane. We found that Arch::GFP is localized to the cell membrane, but a very intense GFP signal also occurs in intracellular vesicular structures. Translocation of Arch from such vesicles to the plasma membrane might be involved in creating the delay in acquiring the silencing function. Although the GFP segment, by promoting cytoplasmic localization, might affect incorporation of tagged-proteins into the plasma membrane, we found that the removal of GFP from Arch::GFP did not drastically alter either the time course or the dependence on light intensity concerning the responsiveness of Arch-induced worms.

Another possibility that would account for the delay is that newly synthesized Arch might undergo a conformation change, or post-translational modifications before becoming effective. Our finding that ATR can be incorporated into preexisting Arch within 1.5 hours argues against the idea that the delay represents a period needed for incorporation of ATR. A previous *in vitro* study showing that ATR does not affect the rate-limiting step of bacteriorhodopsin folding [25] also supports this notion. We have also shown that Arch, once having incorporated ATR, remains functional in the absence of ambient ATR. This is consistent with the fact that retinoids keep binding to bacteriorhodopsin after light-driven isomerization [26].

### Comparison with NpHR

NpHR, an inward chloride pump, is currently the most widely used optogenetic tool for silencing neurons. A previous *in vitro* study showed that Arch can silence neurons at a lower light intensity than NpHR [11]. In a previous study, the green light with an intensity of 4.4 mW/mm^2^ or 10.2 mW/mm^2^ were used for silencing of *C. elegans* body wall muscle cells expressing NpHR [7]. With Arch driven by the same *myo-3* promoter, we found that the light intensity of 8.3 mW/mm^2^ was sufficient to stop locomotion of about half of the animals. While we have not compared the light dependence of silencing effects of these two pumps directly, Arch appears to have hyperpolarizing effects comparable to NpHR on *C. elegans* cells. The resistance to inactivation during prolonged illumination makes Arch a useful alternative to NpHR in optogenetic studies of *C. elegans*.

## Materials and Methods

### *C. elegans* Culture Conditions

Worm strains expressing Arch were grown on Nematode Growth Medium (NGM) plates [27] seeded with solution of *Escherichia coli* OP50 and 500 µM all-trans-retinal (ATR) (Sigma-Aldrich, USA). Animals were maintained at 20°C in the dark unless indicated otherwise. *C. elegans* wild-type strain N2 and CB307 *unc-47(e307)* III were used.

### Plasmid Construction

*Arch::gfp* cDNA was amplified from AAV-FLEX-Arch-GFP [11] (Addgene, Cambridge, MA, USA) by PCR using 5′AAGGTACCGGTAGAAAAAATGGACCCCATCGCTCTGAGG3’ and 5′TTGGTACCTTACTTGTACAGCTCGTCCA3’ as primers, and was cloned into pBluescriptII (pBS, Strategene, USA) with restriction enzymes *Kpn*I/*Sac*I. To generate *hsp-16.2p::Arch::gfp* (pOKA020), *Arch::gfp* cDNA cut out from pBS cDNA was cloned into the *Kpn*I/*Sac*I site of pPD49.78 containing the *hsp-16.2* promoter [28].

The other plasmids were made using the TOPO cloning kit and the Gateway system (Invitrogen, USA). To generate the entry clone pENTR/D carrying *Arch::gfp* (pOKA002), *Arch::gfp* cDNA was PCR-amplified by using 5′GGTACCGGTAGAAAAAATGGACCCCTCGCTCTGCAGGCTGG3’ and 5′ATGGACCCCTCGCTCTGC3’, and 5′ AAGGTACCTTACTTGTACAGCTCGTC3’, and then was cloned into pENTR/D by TOPO reaction to generate pOKA001 and pOKA002, respectively (following the manufacturer’s instructions). pOKA002 was recombined by LR reaction into destination vectors pDEST-*F25B3.3p* and pDEST-*aex-3p* [29], and pOKA051 was recombined into pDEST-*myo-3p* [29] (gift from Hidehito Kuroyanagi). To make pENTR/D Arch (pOKA003), Arch cDNA was amplified using 5′GGTACCGGTAGAAAAAATGGACCCCTCGCTCTGCAGGCTGG3’ and 5′TTATGCTACTACCGGTCGGT3’, and then was cloned into pENTR/D by TOPO reaction. pOKA003 was recombined into pDEST-*hsp-16.2*p [29] by LR reaction (gift from Hidehito Kuroyanagi).

Destination vectors containing neuron-subset-specific promoters were constructed by inserting the PCR-amplified genomic fragments into the *Sph*I site of pDEST-PL (gift from Hidehito Kuroyanagi) using the following primers:

for pDEST-*del-1p*,

5′AAGCATGCTCAGTATCATTGATTATTATCATTAGTTCG3’ and.

5′AAGCATGCACCCCATCTTTTCAAAAACTTCGGCTTC3’;

for pDEST-*unc-47p*,

5′AAGCATGCCTGGGTAAACCTATCATACGAAACTGC3’ and.

5′AAGCATGCTCTAGACTGTAATGAAATAAATGTGACGCTGTCG3’;

for pDEST-*acr-5p*,

5′AAGCATGCGGTATACTTATCGGTGAGTTGAATATGCACC3’ and.

5′AAGCATGCTCTAGAGCTGAAAATTGTTTTTAAAGCATTGAAACTGG3’.

To generate *del-1p::Arch::gfp, unc-47p::Arch::gfp, acr-5p::Arch::gfp,* and *unc-4p::Arch::gfp,* pOKA002 was recombined into pDEST-*del-1p*, pDEST-*unc-47p*, pDEST- *acr-5p*, and pDEST-*unc-4p* (gift from Hidehito Kuroyanagi) by LR reaction respectively.

### Transgenic Strains

Transgenic animals were generated by microinjection of DNA into the distal arms of gonads of N2 hermaphrodites as previously described [30]. pOKA049 (*myo-3p::Arch::gfp*, 70 ng/µl) and pRF4 (*rol-6d,* 400 ng/µl), which is a transgenic marker conferring the Roller phenotype, were injected together into N2 worms to create the line *ncEx3031[pOKA049 (myo-3p::Arch::gfp), pRF4 (rol-6d)]*; pOKA052 (*F25B3.3p::Arch::gfp*, 170 ng/µl) and pRF4 (600 ng/µl) to create *ncEx3034[pOKA052(F25B3.3p::Arch::gfp), pRF4 (rol-6d)]*; pOKA048 (*aex-3p::Arch::gfp*, 160 ng/µl) and pRF4 (600 ng/µl) to create *ncEx3026[pOKA048 (aex-3p::Arch::gfp), pRF4 (rol-6d)]*; pOKA020 (*hsp-16.2p::Arch::gfp*, 75 ng/µl) and pRF4 (125 ng/µl) to create *ncEx3002, ncEx3003* and *ncEx3004[pOKA020 (hsp-16.2p::Arch::gfp), pRF4 (rol-6d)]*; pOKA056 (*hsp-16.2p::Arch*, 110.8 ng/µl), pPD49.78 GFP (*hsp16-2p::gfp*, 140 ng/µl) and pRF4 (230 ng/µl) to create *ncEx3039, ncEx3040* and *ncEx3041[pOKA056 (hsp-16.2p::Arch), pPD49.78 gfp (hsp16-2p::gfp), pRF4 (rol-6d)]*.

Non-roller transgenic animals were generated by using pCFJ90 (*myo-2p::mCherry*) which drives expression of mCherry in the pharyngeal muscles, as a transgenic marker. We injected into N2 worms pOKA073 (*del-1p::Arch::gfp*, (200 ng/µl), pCFJ90 (10 ng/µl) and pBluescript (500 ng/µl) to create *ncEx2365[pOKA073(del-1p::Arch::gfp), pCFJ90* (*myo-2p::mCherry*)*]*;

pOKA074 (*unc-47p::Arch::gfp*, (200 ng/µl), pCFJ90 (10 ng/µl) and pBluescript (500 ng/µl) to create *ncEx2351[pOKA074(unc-47p::Arch::gfp), pCFJ90* (*myo-2p::mCherry*)*]*;

pOKA075 (*acr-5p::Arch::gfp*, (200 ng/µl), pCFJ90 (10 ng/µl) and pBluescript (500 ng/µl) to create *ncEx2371[pOKA075(acr-5p::Arch::gfp), pCFJ90* (*myo-2p::mCherry*)*]*; and pOKA068 (*unc-4p::Arch::gfp*, (200 ng/µl), pCFJ90 (10 ng/µl) and pBluescript (500 ng/µl) to create *ncEx3068[pOKA068(unc-4p::Arch::gfp), pCFJ90* (*myo-2p::mCherry*)*]*.

Since all the strains expressing Arch::GFP used in this study carried a transgene in the form of an extra-chromosomal array, the expression level of Arch::GFP varied among animals of a single transgenic line. It also varied among cells in a transgenic animal. Thus we did not observe the expression of Arch::GFP in all body wall muscle cells of an *ncEx3031[myo-3p::Arch::gfp]* animal, nor in all neurons of an *ncEx3034 [F25B3.3p::Arch::gfp]* animal or an *ncEx3026 [aex-3p::Arch::gfp]* animal.

### Heat-shock Induction of Arch

All worms tested were F_1_ roller progeny of P_0_ roller adults picked onto NGM plates with or without ATR three days before experiments. Late L4 larvae were transferred to NGM plates with or without ATR. The culture plates containing worms were placed in a bacteria culture incubator at 37°C for 30 minutes, and then were returned to the worm incubator at 20°C. The room was maintained at 25°C.

### Microscopic Observation of GFP-expressing Worms

For observation of GFP fluorescence, a fluorescence stereo microscope SZX12 (Olympus, Tokyo, Japan) with a 100W HBO mercury lamp (Osram, Munich, Germany), and a fluorescence microscope Axioplan 2 (Zeiss, Oberkochen, Germany) with filter set #10 for GFP with a 100W HBO mercury lamp (Osram, Munich, Germany) were used. Images were acquired with a digital CCD camera Cool SNAP HQ^2^ (Photometrics, Tucson, AZ, USA) using PM Capture Pro software (Nippon Roper, Japan), and processed using ImageJ public domain software.

### Locomotion Assay Under Illumination

For locomotion assays to examine dependence on light intensity, 5 animals were transferred to a new 30mm NGM plate supplemented with ATR. Worms exhibiting the Roller phenotype or expressing mCherry- in the pharynx were chosen as transgenic animals. With strains in which the Arch::GFP expression was driven by tissue-specific promoters, animals with intense GFP signals were further selected for the assay. Animals were observed under a fluorescence stereo microscope (SZX12), and were illuminated with a 100W HBO mercury lamp through an excitation filter for Red Fluorescent Protein DsRed (545±10 nm, Olympus, Tokyo, Japan). Images of moving animals were recorded using a digital video camera G2-HM570B (JVC, Yokohama, Japan) mounted on the microscope. In most experiments, worms were used only once for a single series of illumination experiments with varying light intensities (from 0.1 to 17 mW/mm^2^), and were then discarded unless otherwise indicated.

For locomotion assays of animals expressing Arch::GFP in motor neuron subsets, animals were observed under a fluorescence stereo microscope (MVX10), and were illuminated with a 100W HBO mercury lamp through the filter set U-MRFPHQ (Olympus, Tokyo, Japan) with the light intensity of 14.7 mW/mm^2^ at 550 nm. Locomotory defects of freely moving animals were scored through observation of each animal for 5 min. At least 30 animals were examined for each transgenic line. The response to gentle touch was assayed as described by Hart [31]. Briefly, for examining worm’s responses to gentle touch, animals were touched by stroking a hair across the worm’s body at different positions: just behind the pharynx for anterior touch, and just anterior to the anus for posterior touch. For examining the response to harsh touch, animals were prodded with a platinum wire. At least 30 animals were examined for each transgenic line, and each animal was examined 5 times.

In time course analyses of silencing activity of *ncEx3003* after heat-shock induction, animals were used only once for each time point. In experiments comparing the time course for heat-shock-induced Arch::GFP in *ncEx3002, ncEx3003* and *ncEx3004* animals, and Arch in *ncEx3039, ncEx3040* and *ncEx3041* animals, worms were used for a single series of illumination experiments at various time points (3,6,9,12,24 and 48 hours after heat shock). After observation at each time point, plates were returned to the worm incubator at 20°C, and before the next observation, plates were left for 30 minutes at 25°C.

### Measurement of Light Intensity

Light intensity at 550 nm, corresponding to the Arch absorption maximum, was measured at the object plane using an optical power meter (#3664, Hioki, Ueda, Japan) with an optical sensor (#9742, Hioki, Ueda, Japan). Various intensities of illumination were implemented by changing the magnification of the objective lens of the microscope. To calculate the intensity of illumination, we measured the radius of the illuminated area, calculated the area, and then divided the total light intensity by the area.

## Supporting Information

Figure S1 **Expression of Arch::GFP in** ***C. elegans*** **driven by various promoters.** (A) A fluorescent micrograph of an *nc2351Ex[unc-47p::Arch::gfp]* animal. Arch::GFP is expressed in D-type motor neurons (VD, DD) (arrow). (B) Expression of Arch::GFP in an *nc3068Ex[unc-4p::Arch::gfp]* animal. Arch::GFP is expressed in A-type motor neurons (arrow). (C) Expression of Arch::GFP in an *nc2371Ex[acr-5p::Arch::gfp]* animal. Arch::GFP is expressed in B-type motor neurons (arrow). (D) Expression of Arch::GFP in an *nc2365Ex[del-1p::Arch::gfp]* animal. Arch::GFP is expressed in VA and VB motor neurons (arrow). The fluorescence of mCherry expressed in the pharynx is also detected. Scale bar  = 100 µm. Anterior is toward the right and dorsal is up.
(TIF)

Figure S2 **Defects in the locomotory behavior caused by silencing subsets of motor neurons in strains carrying the** ***rol-6d gene*** **.** (A-C) Locomotory behavior of worms expressing Arch in motor neurons subsets. Arch::GFP was expressed in D-type (VD, DD) and VA +VB motor neurons in *ncEx2311[unc-47p::Arch::gfp; rol-6d]* and *ncEx2322 [del-1p::Arch::gfp; rol-6d]* animals exhibiting the Roller phenotype. To evaluate differences of animal’s behavior statistically, we used Fisher's exact test for the locomotion assay of freely moving animals (A), and Student’s *t* test for the touch response assay (B, C). For all transgenic strains, animals behaviors under green light illumination (ON = open box) and those without illumination (OFF = filled box) differed significantly (p<0.001). Error bars indicate ±SEM. (A) Forward (F) and backward movement (B) was scored in worms moving freely. Percentage of animals exhibiting the “Class 3 (severe)”, “Class2 (mild)” and “Class1 (no)” phenotype in locomotory behaviors when they were illuminated with green light is shown. (B) Forward movement (F) to gentle posterior touch and backward (B) movement to gentle anterior touch were scored. Responses out of five touches are shown. (C) Forward (F) and backward movement (B) to harsh touch was scored, and was shown additively in each bar. Responses out of five touches are shown.
(TIF)
