# Supplementary figures and images for: Optical Silencing of C. elegans Cells with Arch Proton Pump
Source: PLoS One. 2012 May 21;7(5):e35370. doi: 10.1371/journal.pone.0035370 (PMC3357435; doi:10.1371/journal.pone.0035370)

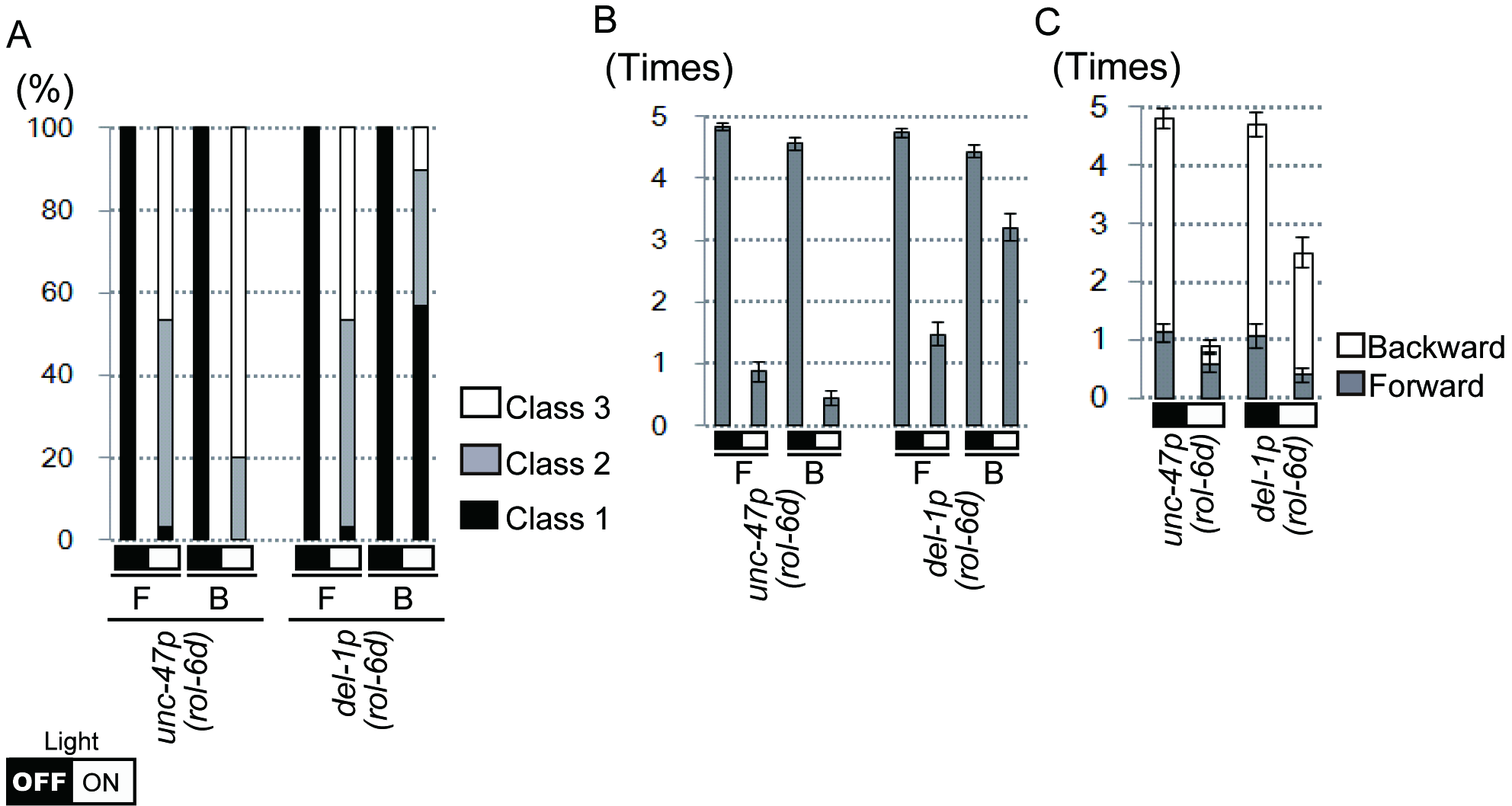

Supplement: Figure S1 — Expression of Arch::GFP in C. elegans driven by various promoters. (A) A fluorescent micrograph of an nc2351Ex[unc-47p::Arch::gfp] animal. Arch::GFP is expressed in D-type motor neurons (VD, DD) (arrow). (B) Expression of Arch::GFP in an nc3068Ex[unc-4p::Arch::gfp] animal. Arch::GFP is expressed in A-type motor neurons (arrow). (C) Expression of Arch::GFP in an nc2371Ex[acr-5p::Arch::gfp] animal. Arch::GFP is expressed in B-type motor neurons (arrow). (D) Expression of Arch::GFP in an nc2365Ex[del-1p::Arch::gfp] animal. Arch::GFP is expressed in VA and VB motor neurons (arrow). The fluorescence of mCherry expressed in the pharynx is also detected. Scale bar = 100 µm. Anterior is toward the right and dorsal is up. (TIF) [file pone.0035370.s001.tif]

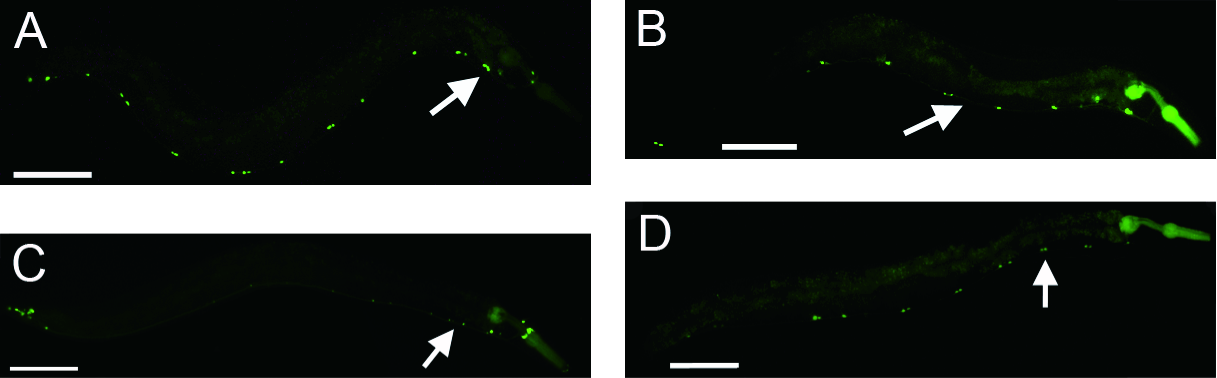

Supplement: Figure S2 — Defects in the locomotory behavior caused by silencing subsets of motor neurons in strains carrying the rol-6d gene . (A-C) Locomotory behavior of worms expressing Arch in motor neurons subsets. Arch::GFP was expressed in D-type (VD, DD) and VA +VB motor neurons in ncEx2311[unc-47p::Arch::gfp; rol-6d] and ncEx2322 [del-1p::Arch::gfp; rol-6d] animals exhibiting the Roller phenotype. To evaluate differences of animal’s behavior statistically, we used Fisher's exact test for the locomotion assay of freely moving animals (A), and Student’s t test for the touch response assay (B, C). For all transgenic strains, animals behaviors under green light illumination (ON = open box) and those without illumination (OFF = filled box) differed significantly (p<0.001). Error bars indicate ±SEM. (A) Forward (F) and backward movement (B) was scored in worms moving freely. Percentage of animals exhibiting the “Class 3 (severe)”, “Class2 (mild)” and “Class1 (no)” phenotype in locomotory behaviors when they were illuminated with green light is shown. (B) Forward movement (F) to gentle posterior touch and backward (B) movement to gentle anterior touch were scored. Responses out of five touches are shown. (C) Forward (F) and backward movement (B) to harsh touch was scored, and was shown additively in each bar. Responses out of five touches are shown. (TIF) [file pone.0035370.s002.tif]
